# ursaPGx: a new R package to annotate pharmacogenetic star alleles using phased whole-genome sequencing data

**DOI:** 10.3389/fbinf.2024.1351620

**Published:** 2024-03-12

**Authors:** Gennaro Calendo, Dara Kusic, Jozef Madzo, Neda Gharani, Laura Scheinfeldt

**Affiliations:** ^1^ Coriell Institute for Medical Research, Camden, NJ, United States; ^2^ Cooper Medical School of Rowan University, Camden, NJ, United States; ^3^ Gharani Consulting Limited, London, United Kingdom

**Keywords:** R, pharmacogenetic, star allele, software, annotation

## Abstract

Long-read sequencing technologies offer new opportunities to generate high-confidence phased whole-genome sequencing data for robust pharmacogenetic annotation. Here, we describe a new user-friendly R package, ursaPGx, designed to accept multi-sample phased whole-genome sequencing data VCF input files and output star allele annotations for pharmacogenes annotated in PharmVar.

## 1 Introduction

Pharmacogenomics (PGx) benefits medication management (Gharani et al., 2013; Dunnenberger et al., 2015; Relling and Evans, 2015; Zhang et al., 2015; Bush et al., 2016; Relling et al., 2017; Bank et al., 2018); however, pharmacogenetic annotation is often quite complex (Supplementary Figure S1). Functional PGx annotation and corresponding clinical PGx recommendations rely on star (*) allele annotation (Caudle et al., 2014; Kalman et al., 2016); star alleles are often defined by more than one genetic variant (Gaedigk et al., 2018; Gaedigk et al., 2020; Gaedigk et al., 2021); when the star allele-defining variants are heterozygous, phased haplotype information is needed to resolve the annotation. In addition, annotations may change over time as new variants are characterized and incorporated into clinical PGx recommendations. Many resources and off-the-shelf tools are available to support researchers and clinicians interested in PGx annotation. Several tools are well-suited for the PGx annotation of unphased data (e.g., StellarPGx and Stargazer (Lee et al., 2019; Twesigomwe et al., 2021)), and tools such as PharmCAT, while not computationally streamlined for multi-sample annotation, go a step further to incorporate clinical recommendations into the software output (Sangkuhl et al., 2020).

New long-read sequencing technologies offer opportunities to generate high-confidence phased whole-genome sequencing (WGS) data for robust PGx annotation. Here, we describe ursaPGx, an R package designed to complement existing tools that leverages phased whole-genome sequencing data for PGx annotation. ursaPGx is designed to run on a typical laptop using multi-sample, phased, WGS VCF files and provides an output table of star allele annotations for selected pharmacogenes annotated in PharmVar.

## 2 Materials and methods

### 2.1 Samples

Phased multi-sample VCF files were downloaded for each of the star allele containing chromosomes from the 1000 Genomes Project. These VCF files were generated by the New York Genome Center for 3,202 1000 Genomes Project samples by aligning the 30× WGS reads to GRCh38 and performing SNV and INDEL variant calling, as described in Byrska-Bishop et al. (2022).

### 2.2 Benchmark data

The accuracy of the star allele calling algorithm of ursaPGx was benchmarked against the next-generation sequencing consensus calls generated by the Genetic Reference and Testing Material Coordination Program (GeT-RM) for *CYP2C8*, *CYP2C9,* and *CYP2C19*, which combined the output of Astrolabe (Twist et al., 2016), Stargazer (Lee et al., 2019), and Aldy (Numanagic et al., 2018) across investigator groups to generate a uniform diplotype call for each of the 137 samples included in their study (Gaedigk et al., 2022), of which 87 also have 30× WGS data (Byrska-Bishop et al., 2022). *CYP2D6* calls generated by ursaPGx’s implementation of Cyrius were benchmarked against calls generated by Chen et al. (2021).

### 2.3 Implementation and algorithm description

**FIGURE 1:**
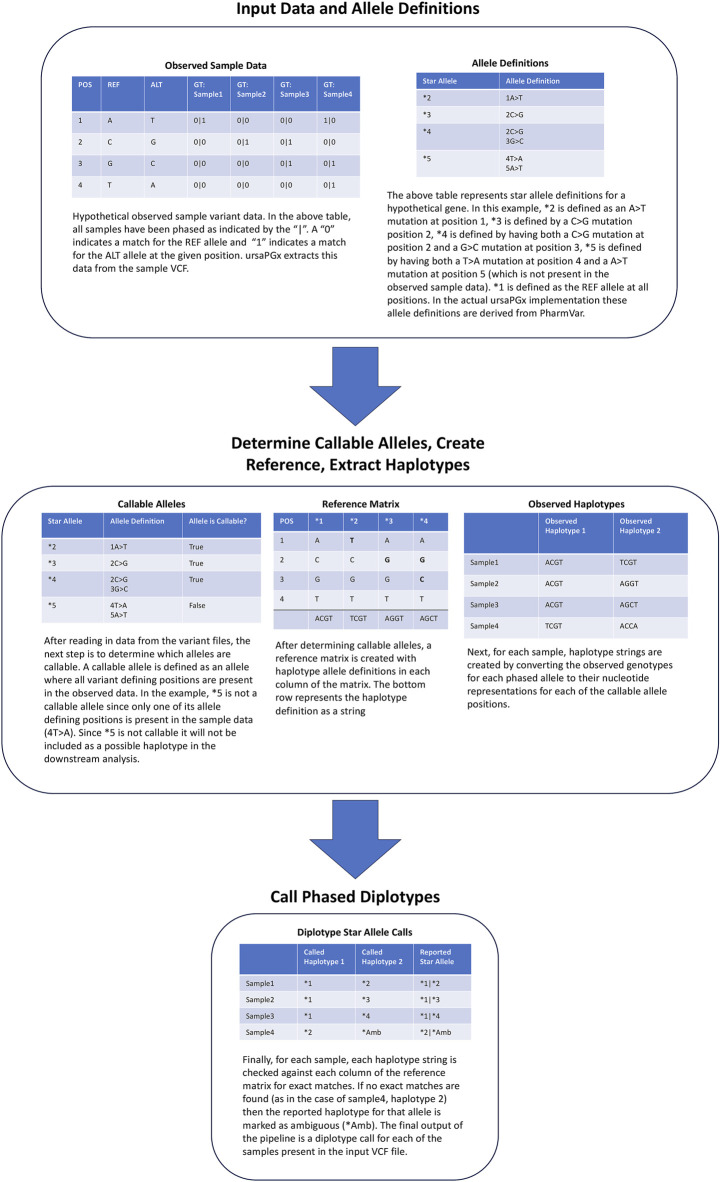
ursaPGx pipeline overview and example annotation. The figure illustrates the main steps of the ursaPGx annotation pipeline along with a toy annotation example for four samples and a hypothetical pharmacogene gene. ursaPGx takes phased VCF files as input and, along with PharmVar allele definitions, extracts haplotype data from each sample and performs exact matching against each definition. The final reported output is a diplotype call for each sample. For any haplotype that is not found to have an exact match to a known allele definition, “ambiguous” (*Amb) is assigned.

Users may choose any phased WGS VCF file of interest for use as input to ursaPGx. ursaPGx assigns phased diplotype calls from single-sample or multi-sample indexed VCF files using publicly available star allele definitions from PharmVar (Gaedigk et al., 2018; Gaedigk et al., 2020; Gaedigk et al., 2021). An overview of the annotation algorithm is shown in Figure 1. First, for a given pharmacogene, star allele-defining positions are used to extract genotype data for all samples in the VCF. Next, the extracted positions are checked against each PharmVar haplotype definition to determine ‘callable’ alleles. In this context, a callable allele is defined as a haplotype definition where all allele-defining variants are present in the sample VCF. Downstream analysis is then limited to the set of callable alleles. The set of callable alleles is then used to generate a genomic position by a haplotype definition reference matrix. The cells of the reference matrix contain the nucleotide which defines the given haplotype for each of the positions present in the sample VCF. Positions that are not part of a given haplotype definition are filled with the reference nucleotide for the position. Using this reference matrix allows ursaPGx to disambiguate star allele definitions such as CYP2C19*2 and CYP2C19*35, which share the same core allele definitions (CYP2C19*2, non-reference alleles for rs4244285, rs12769205, and rs3758581; CYP2C19*35, non-reference alleles for rs12769205 and rs3758581) and, therefore, must be distinguished using a SNV unique to CYP2C19*2 (rs4244285)*.* After constructing the reference matrix, genotype calls are converted to their nucleotide representation and split into haplotype strings for each sample. For each sample, each haplotype string is checked for exact matches against all columns of the reference matrix. All exact matches to the reference for each sample haplotype string are reported for each sample. If no exact matches occur, then the haplotype call for that sample is reported as ambiguous (*Amb). Haplotype calls for each sample are then combined to form a single diplotype call for the given pharmacogene for each sample included in the VCF.

*CYP2D6* star allele calling in ursaPGx is performed with a modified version of the Illumina *CYP2D6* star allele caller Cyrius, designed to function in R. The *CYP2D6* haplotype calling algorithm implemented in Cyrius is fully described in Chen et al. (2021). In brief, Cyrius uses WGS BAM files to estimate the total number of copies of *CYP2D6* and *CYP2D7*, determines the number of complete *CYP2D6* and hybrid genes, and uses these to estimate SVs impacting the *CYP2D6* annotation*.* Cyrius then performs small variant calling for star allele-defining positions and derives an estimate of their copy number, and then matches these calls and SVs against star allele definitions from PharmVar (7/15/2020) to produce final diplotype calls for each sample.

### 2.4 Software and requirements

ursaPGx is a freely available and open source package implemented in the R programming language (R Core Team, 2020) and utilizes the *VariantAnnotation* package (Obenchain et al., 2014) from the Bioconductor project to provide a consistent interface with existing R packages for the analysis of genetic variant data. Star allele definitions in VCF format are downloaded from PharmVar (current version 5.2.13) and parsed into R objects. All package code and analysis scripts are hosted on GitHub (https://github.com/coriell-research/ursaPGx).

ursaPGx is designed to run on a personal laptop. Star allele calling for all 3,202 1000 Genomes Project samples for all 12 pharmacogenes takes ∼45 s on a 3.7 GHz 6-Core Intel Core i5 iMac device. Cyrius *CYP2D6* calling implemented in ursaPGx takes ∼4 s per sample BAM.

## 3 Results

*CYP2C8*, *CYP2C9*, and *CYP2C19* concordance was assessed for samples with matching IDs from the 30× WGS data in the GeT-RM benchmarking datasets (87/137) (Gaedigk et al., 2022). *CYP2D6* concordance was tested against diplotype calls from Chen et al. (2021) to ensure accuracy of the Cyrius implementation within ursaPGx. Diplotype calls produced by ursaPGx were found to be highly consistent with those generated by GeT-RM for all three benchmarked pharmacogenes (Table 1, Supplementary Table S1). For the 87 samples with matching IDs between the 1000 Genomes Project 30× WGS data and the GeT-RM NGS consensus benchmarking data, *CYP2C8* was found to be perfectly concordant (Gaedigk et al., 2022). For *CYP2C19*, one subject sample (NA19122) was reported as *2|*Amb, according to ursaPGx whereas the GeT-RM consensus call for this sample was reported as *2/*35 (Gaedigk et al., 2022). In the phased 30× WGS dataset, one haplotype was an exact match for CYP2C19*2 but the other haplotype had no exact match to any PharmVar definition (Gaedigk et al., 2022). Assuming accurate phasing of the input 30× WGS dataset, ursaPGx reports the inexact match as ambiguous for this sample.

**TABLE 1:** Concordance of ursaPGx diplotype calls with benchmarking datasets.

Gene	Concordance	Benchmarking data
*CYP2C8*	1.00 (87/87)	GeT-RM (Gaedigk et al., 2022)
*CYP2C9*	0.97 (84/87)	GeT-RM (Gaedigk et al., 2022)
*CYP2C19*	0.99 (86/87)	GeT-RM (Gaedigk et al., 2022)
*CYP2D6*	0.99 (2502/2504)	Cyrius (Chen et al., 2021)

For *CYP2C9*, three samples were found to be discordant between ursaPGx and GeT-RM reported consensus calls (Gaedigk et al., 2022). Two of the subject samples with discordant *CYP2C9* calls, NA19143 and NA19213, were annotated as *1/*6 by GeT-RM, whereas ursaPGx assigned these samples as *1|*1 (Gaedigk et al., 2022). Because the CYP2C9*6 defining variant (rs9332131) is not present in the phased 30× WGS dataset, CYP2C9*6 is not included as a callable allele by ursaPGx and is, thus, not reported for these samples. One subject sample, HG01190, was assigned as *61|*1 by ursaPGx, whereas GeT-RM reported the diplotype as *2/*61 (Gaedigk et al., 2022). However, this sample was found to be inconsistently annotated across laboratories in the GeT-RM benchmarking data with a minority subset of three of the annotation approaches assigning *1/*61 (Gaedigk et al., 2022). Additionally, in the 30× WGS dataset, rs1799853 and rs202201137 are both heterozygous, and the non-reference allele for rs1799853 (CYP2C9*2) is on the same phased chromosome as the rs202201137 non-reference allele (presence of both non-reference alleles on the same haplotype defines the *61 variant according to PharmVar). Given the phase information from the 30× WGS dataset, *61|*1 is the diplotype that is most consistent with the observed data for this sample.

Since Cyrius has already been shown to produce highly accurate *CYP2D6* star allele calls (Chen et al., 2021), we benchmarked ursaPGx’s implementation of Cyrius against the 2,504 Phase 3 1000 Genomes Project sample data (Genomes Project et al., 2015) analyzed in the Cyrius publication in order to ensure that changes made to Cyrius, which were needed to port the software package to R, were consistent with the original Cyrius implementation (Supplementary Table S2). Of the 2,504 samples, 2,502 samples were found to be exact matches with the Cyrius reported results (Chen et al., 2021). For the two discordant samples, NA18611 and HG02490, ursaPGx reported diplotype calls for these samples (*10/*2 and *2/*33, respectively), whereas the Cyrius benchmark did not assign a diplotype for these samples (Chen et al., 2021). This discrepancy is likely due to differences in BAM file input and downstream processing used in the 1000 Genomes Project NYGC 30× WGS data versus the WGS dataset used in the Cyrius publication (Chen et al., 2021).

## 4 Discussion

Here, we describe a new pharmacogenetic annotation tool, ursaPGx, that is designed to complement existing tools by leveraging multi-sample phased WGS data and PharmVar annotations. ursaPGx is implemented as an efficient and user-friendly R package that provides a simple interface for assigning star allele diplotypes to samples for PharmVar-annotated genes including *CYP2D6*, by integrating the Cyrius *CYP2D6* star allele caller (Chen et al., 2021). Indeed, we recently employed ursaPGx to annotate a large and diverse whole-genome sequencing dataset (Gharani et al., 2024). This analysis served as an illustrative use case of the new tool and provided examples of the utility of pharmacogenetic annotation in a large and diverse collection of biospecimens.

Being implemented as an R package, ursaPGx offers easy dependency management and simple installation instructions. Because of its simple API, it is relatively easy for users with little computational skill to generate star allele calls. However, since ursaPGx also inherits much of its functionality from existing Bioconductor classes and methods, more advanced users can inspect and manipulate every step of the star allele calling pipeline when needed. ursaPGx has also been designed to be compatible with future updates to the PharmVar database. Allele definitions are extracted directly from PharmVar database VCF definition files, ensuring future versions of the package can use the most up-to-date versions of the PharmVar allele definitions.

Our benchmarking analysis demonstrated high concordance, 100%, 97% and 99%, respectively, for the three overlapping pharmacogenes, *CYP2C8*, *CYP2C9*, and *CYP2C19* included in the most recent GeT-RM report (Gaedigk et al., 2022). Two of the discordant samples for *CYP2C9* result from a star allele-defining variant (*6) that is present in the GeT-RM dataset but not occurring in the 30× WGS 1000 Genomes Project dataset used to benchmark ursaPGx (Gaedigk et al., 2022). The third discordant *CYP2C9* sample (HG01190) results presumably from differences in phasing and variant calling results (Gaedigk et al., 2022). Finally, as detailed in the Methods section above, when no perfect match to any PharmVar defined haplotype occurs, the ursaPGx output will be “*Amb,” and this implementation approach explains the single discordant *CYP2C19* sample, NA19122.

As with any annotation approach, ursaPGx includes several limitations. First and foremost, any error or missing variants in the input VCF file will propagate into errors in annotation. Similarly, any errors or uncertainty in phase will propagate into annotation errors, particularly when heterozygotes are phased incorrectly. In addition, our annotation approach is limited to the pharmacogenes annotated in PharmVar (Gaedigk et al., 2018; Gaedigk et al., 2020; Gaedigk et al., 2021) and requires already phased input data. This annotation choice is specifically designed to take advantage of increasingly common long-read WGS datasets, such as the data being generated by the Human Pangenome Reference Consortium (Liao et al., 2023).

## Data Availability

Publicly available datasets were analyzed in this study. These data can be found at: NYGC WGS data (VCF files): https://ftp.1000genomes.ebi.ac.uk/vol1/ftp/data_collections/1000G_2504_high_coverage/working/20220422_3202_phased_SNV_INDEL_SV/). The version we used for the current study was last modified on 2022-11-14 08:33. All package code and analysis scripts are hosted on GitHub: https://github.com/coriell-research/ursaPGx.
