# Supplementary figures and images for: ursaPGx: a new R package to annotate pharmacogenetic star alleles using phased whole-genome sequencing data
Source: Front Bioinform. 2024 Mar 12;4:1351620. doi: 10.3389/fbinf.2024.1351620 (PMC10963438; doi:10.3389/fbinf.2024.1351620)

## Slide 1
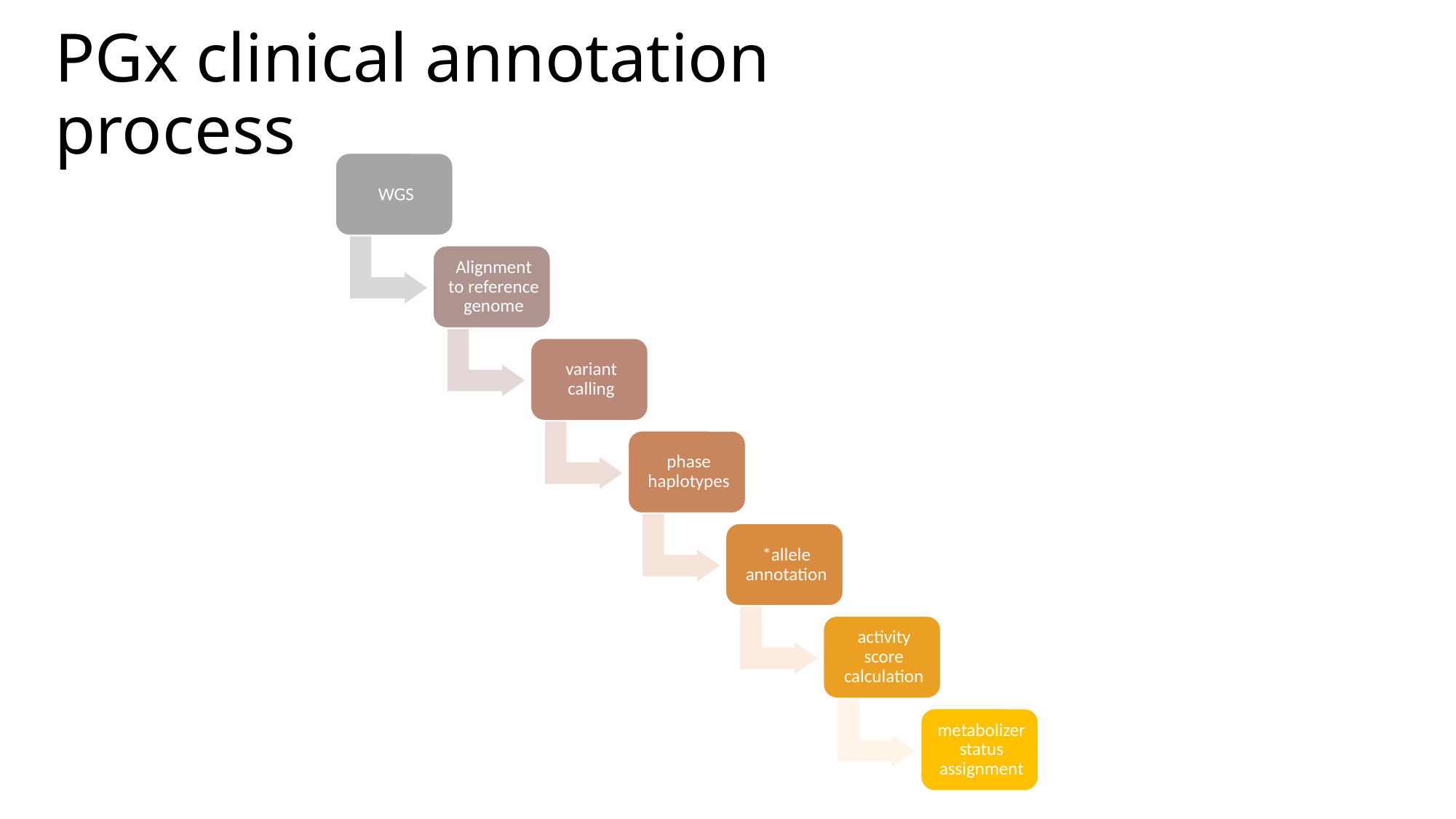

# PGx clinical annotation process

Supplement: Supplementary file 2 [file Presentation1.PPTX]
